# The respiratory depressant effects of mitragynine are limited by its conversion to 7‐OH mitragynine

**DOI:** 10.1111/bph.15832

**Published:** 2022-03-30

**Authors:** Rob Hill, Andrew C. Kruegel, Jonathan A. Javitch, J. Robert Lane, Meritxell Canals

**Affiliations:** ^1^ Division of Physiology, Pharmacology and Neuroscience, School of Life Sciences, Queen's Medical Centre University of Nottingham Nottingham UK; ^2^ Centre of Membrane Proteins and Receptors Universities of Nottingham and Birmingham Midlands UK; ^3^ Department of Chemistry Columbia University New York New York USA; ^4^ Departments of Psychiatry and Molecular Pharmacology and Therapeutics Columbia University Vagelos College of Physicians and Surgeons New York New York USA; ^5^ Division of Molecular Therapeutics New York State Psychiatric Institute New York New York USA

## Abstract

**Background and Purpose:**

Mitragynine, the major alkaloid in *Mitragyna speciosa* (kratom), is a partial agonist at the μ opioid receptor. CYP3A‐dependent oxidation of mitragynine yields the metabolite 7‐OH mitragynine, a more efficacious μ receptor agonist. While both mitragynine and 7‐OH mitragynine can induce anti‐nociception in mice, recent evidence suggests that 7‐OH mitragynine formed as a metabolite is sufficient to explain the anti‐nociceptive effects of mitragynine. However, the ability of 7‐OH mitragynine to induce μ receptor‐dependent respiratory depression has not yet been studied.

**Experimental Approach:**

Respiration was measured in awake, freely moving, male CD‐1 mice, using whole body plethysmography. Anti‐nociception was measured using the hot plate assay. Morphine, mitragynine, 7‐OH mitragynine and the CYP3A inhibitor ketoconazole were administered orally.

**Key Results:**

The respiratory depressant effects of mitragynine showed a ceiling effect, whereby doses higher than 10 mg·kg^−1^ produced the same level of effect. In contrast, 7‐OH mitragynine induced a dose‐dependent effect on mouse respiration. At equi‐depressant doses, both mitragynine and 7‐OH mitragynine induced prolonged anti‐nociception. Inhibition of CYP3A reduced mitragynine‐induced respiratory depression and anti‐nociception without affecting the effects of 7‐OH mitragynine.

**Conclusions and Implications:**

Both the anti‐nociceptive effects and the respiratory depressant effects of mitragynine are partly due to its metabolic conversion to 7‐OH mitragynine. The limiting rate of conversion of mitragynine into its active metabolite results in a built‐in ceiling effect of the mitragynine‐induced respiratory depression. These data suggest that such ‘metabolic saturation’ at high doses may underlie the improved safety profile of mitragynine as an opioid analgesic.

**Abbreviations**
MPE: maximum possible effect
MV: minute volume
OST: opioid substitution therapy

**What is already known**

- Mitragynine oxidation results in the generation of 7‐OH mitragynine, a more efficacious μ receptor agonist.
- The anti‐nociceptive effects of mitragynine are partly mediated by 7‐OH mitragynine.

**What does this study add**

- Mitragynine and 7‐OH mitragynine induced respiratory depression, but only mitragynine showed a ceiling effect.
- The ceiling effect of mitragynine‐induced respiratory depression is due to metabolic saturation of CYP3A enzyme

**What is the clinical significance**

- Metabolic regulation of opioidergic effects offers potential to improve safety profiles of opioid agonists.

## 1 INTRODUCTION

*Mitragyna speciosa*, more commonly known as kratom, is a plant popular in labour‐intensive agricultural communities and native to Southeast Asia (Adkins et al., 2011), where it is mostly prepared as a recreational drink similar to tea. Kratom tea is consumed both for work‐time stimulation, much like caffeinated drinks, and as a relaxation aid (Singh et al., 2016). Over 40 unique alkaloids have been identified in the kratom plant, with mitragynine accounting for over 60% by mass of the alkaloid extracts (Basiliere et al., 2020; Kruegel et al., 2016; Prozialeck et al., 2012; Suhaimi et al., 2016; Takayama, 2004). Mitragynine and its oxidised metabolite 7‐OH mitragynine have recently been shown to bind and activate opioid receptors, displaying higher affinity for the μ opioid receptor subtype (Chakraborty, Uprety, Slocum, et al., 2021; Kruegel et al., 2016; Matsumoto et al., 2004; Shamima et al., 2012; Todd et al., 2020). The anti‐nociceptive effects of both mitragynine and 7‐OH mitragynine were abolished in μ receptor knockout mice, but not in mice lacking either the δ or the κ opioid receptors (Kruegel et al., 2019). The analgesic effects of kratom, together with the low rates of acute toxicity or overdose following its ingestion (Veltri & Grundmann, 2019), have attracted considerable attention in the search for novel, safer opioid‐based analgesics amid the current opioid crisis (Bhowmik et al., 2021; Chakraborty, Diberto, Faouzi, et al., 2021; Chakraborty, Uprety, Daibani, et al., 2021; Gutridge et al., 2020).

Kratom also has been utilised as an aid for recovering opioid users that have previously maintained themselves on abusable opioids, such as heroin, oxycodone and fentanyl, or have been on opioid substitution therapy (OST) such as methadone or suboxone (Grundmann, 2017; Singh et al., 2016; Swogger et al., 2015). The use of kratom as a recovery aid is not limited to Southeast Asia but is increasingly being sought and used in Europe and North America as a preferred recovery aid to existing prescription OST (Grundmann, 2017; Swogger et al., 2015). Importantly, mitragynine has been shown to produce less respiratory depression than codeine and, to our knowledge, no fatal kratom‐induced respiratory depression has been reported, supporting the recent interest in this compound as a potential route to design safer opioids (Kruegel et al., 2016, 2019; Matsumoto et al., 1996; Smith et al., 2019; Suhaimi et al., 2016; Swogger et al., 2015; Varadi et al., 2016; Veltri & Grundmann, 2019).

The observations that mitragynine elicited anti‐nociception upon oral or intraperitoneal administration, but not when administered subcutaneously, suggested the existence of an active metabolite generated through first‐pass metabolism that would be mediating such effects. Recently, 7‐OH mitragynine has been identified as the product of mitragynine conversion by cytochrome P450 3A4 (CYP3A) (Basiliere & Kerrigan, 2020; Chakraborty, Uprety, Slocum, et al., 2021; Kruegel et al., 2019). 7‐OH mitragynine binds to μ receptors, is significantly more efficacious than mitragynine in vitro and has been detected in brain at concentrations sufficient to explain the analgesic effects of its precursor compound (Chakraborty, Uprety, Slocum, et al., 2021; Kruegel et al., 2019). It has also been suggested that, similar to codeine and morphine, the opioidergic actions of mitragynine would depend on its metabolic conversion to 7‐OH mitragynine. This metabolically limited production of the more efficacious compound may offer an avenue to limit the side effect profile of these drugs (Kruegel et al., 2019). However, while there is evidence showing that 7‐OH mitragynine formed as a metabolite is sufficient to explain the anti‐nociceptive effects of mitragynine (Chakraborty, Uprety, Slocum, et al., 2021; Kruegel et al., 2019), the respiratory depressant effects of 7‐OH mitragynine, relative to mitragynine, have not yet been studied.

Here, we have characterised the respiratory depressant and anti‐nociceptive effects of mitragynine and 7‐OH mitragynine in mice. Both drugs were administered orally, as this route demonstrated better bioavailability (Kruegel et al., 2019) and is also the route of consumption in humans. We show that mitragynine‐induced respiratory depression has a ceiling effect, whereby doses above 10 mg·kg^−1^ produce similar responses. In contrast, 7‐OH mitragynine induces respiratory depression in a dose‐dependent manner. Inhibition of CYP3A reduces the respiratory depressant and anti‐nociceptive effects of mitragynine but not those of 7‐OH mitragynine, supporting the hypothesis that the metabolic conversion of mitragynine underlies the built‐in ceiling respiratory effect of this drug.

## 2 METHODS

### 2.1 Animals

All animal care and experimental procedures were performed in accordance with the UK Animals (Scientific Procedures) Act 1986 and the European Communities Council Directive (2010/63/EU) and were approved by ethical review board at the University of Nottingham (project licence P3C7EE0BA). Animal studies are reported in compliance with the ARRIVE guidelines (Percie du Sert et al., 2020) and with the recommendations made by the British Journal of Pharmacology (Lilley et al., 2020).

Male CD‐1 mice (Charles River, UK) weighing approximately 31 ± 1 g were group housed, 3 per cage, in an environment maintained at 22°C, on a reversed 12 h/12 h dark–light cycle with food and water available ad libitum. Experiments were performed in the dark (active) phase. The experimenter was blinded to all drug treatments, both during the experiment and data analysis. Cages of mice were randomly designated to drug treatments, such that all mice in one cage received the same drug treatment in order to minimise social conflict between different drug effects. A total of 250 mice were used in the study.

### 2.2 Mouse respiration

Respiration was measured in freely moving mice using plethysmography chambers (EMKA Technologies, Paris, France) supplied with room air as described previously (Hill et al., 2016, 2018). Mice were habituated to respiratory chambers for 30 min, on the day prior to experimentation. Respiratory parameters were recorded (IOX software ‐ EMKA Technologies, Paris, France) and averaged over 5 min periods.

Data are presented both as minute volume (MV) and as percentage change from the pre‐drug MV baseline, calculated from data for each individual mouse before collating and plotting as a mean. Mice within cohorts may vary in size and this varies their individual MVs, accordingly; data are presented as a percentage change from each mouse's pre‐drug baseline controls for these inherent variations.

### 2.3 Calculation of equi‐effective doses

To determine equi‐effective doses for each opioid, linear and polynomial regressions were fitted to the peak dose response for each drug. Linear regressions were found to produce the best fit for each agonist. Slope equations were used with a set *Y*‐value of 40% (the degree of respiratory depression) to determine the *X*‐axis dose value predicted to induce this level of effect.

### 2.4 Measurement of anti‐nociception

Prior to the day of experimentation, mice were handled onto and off the inactive hot plate a minimum of 5 times; more aggressive or anxious mice were habituated up to a maximum of 10 times. On the day of experimentation, mice were placed on a hot plate at 52.5°C and the latency to exhibit a pain‐like response (defined as paw withdrawal, jumping, paw licking and fluttering of the hind limbs) was measured. A maximum cut‐off time of 20 s was used to prevent tissue damage. For the same reason, measurements were taken no more frequently than every 15 min. Anti‐nociception was quantified and is presented as both latency to response in seconds and calculated as the percentage of maximum possible effect (%MPE), which was calculated as %MPE = [(test latency − control latency) / (20 − control latency)] × 100.

### 2.5 Experimental design and data analysis

Data from previous experiments (Hill et al., 2016, 2018) where respiratory depression or anti‐nociception was measured following acute opioid administration in naïve mice were used to guide a priori power analyses using G*Power (Version 3.1.9). These calculations indicated that *n* = 6 (respiration experiments) or *n* = 10 (anti‐nociception experiments) for each individual group would produce a significant result if an actual effect occurred. GraphPad Prism 8 was used for all statistical analyses. All data are displayed as mean ± SEM. All statistical tests are shown in the figure legends and Table S1. The data and statistical analyses comply with the recommendations of the *British Journal of Pharmacology* on experimental design and analysis in pharmacology (Curtis et al., 2015).

### 2.6 Materials

Morphine hydrochloride (Macfarlan Smith, Edinburgh, UK— a kind gift from Graeme Henderson, University of Bristol, UK) ‐ was dissolved in distilled water for oral administration. Mitragynine was extracted from the dried leaves of M. speciosa and 7‐OH mitragynine was chemically synthesised as previously described (Kruegel et al., 2016). Both drugs were supplied as free base and dissolved by the addition of 3 molar equivalents of acetic acid followed by dilution in de‐ionised water before oral administration. Ketoconazole (Tocris, UK) was dissolved in a 4:16:80 solution of DMSO:Tween80:distilled water for oral administration. All drugs were administered in 0.1 ml volumes and made up freshly on the day of experiment.

### 2.7 Nomenclature of targets and ligands

Key protein targets and ligands in this article are hyperlinked to corresponding entries in http://www.guidetopharmacology.org and are permanently archived in the Concise Guide to PHARMACOLOGY 2021/22 (Alexander, Christopoulos et al., 2021; Alexander, Fabbro et al., 2021).

## 3 RESULTS

### 3.1 Respiratory depressant and anti‐nociceptive effects of mitragynine and 7‐OH mitragynine

**FIGURE 1:**
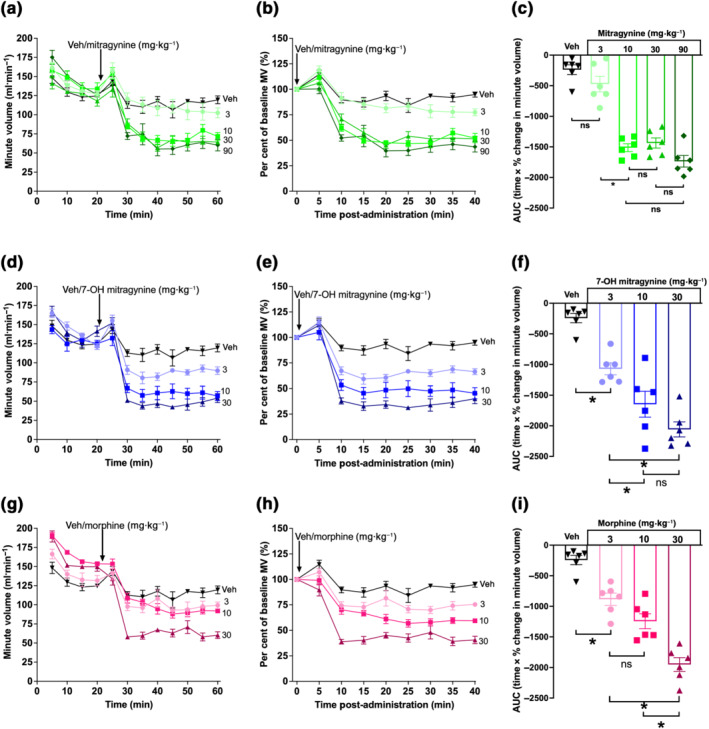
Mitragynine and 7‐OH mitragynine depress respiration in awake mice. (a–c) Mitragynine (10–90 mg·kg^−1^) but not 3 mg·kg^−1^ significantly depressed minute volume (MV) when compared with effects of vehicle alone. There was no significant difference in the degree of depression induced by mitragynine over a similar dose range (10–90 mg·kg^−1^). (d–f) 7‐OH mitragynine significantly depressed mouse MV at all doses in a dose‐dependent manner. (g–i) Morphine significantly depressed mouse MV at all doses in a dose‐dependent manner similar to 7‐OH mitragynine. *N* = 6 for all groups. All drugs administered by oral gavage dosing. In (c), (f) and (i), * *P* < 0.05, significantly different; ns, not significantly different, as indicated; one‐way ANOVA (Kruskal–Wallis) with Dunn's multiple comparison. See Table S1 for full test statistics

The respiratory depressant activity of mitragynine, 7‐OH mitragynine and morphine was assessed using whole body plethysmography in mice breathing air. All compounds were administered orally at increasing doses. Both morphine (3–30 mg·kg^−1^) and 7‐OH mitragynine (3–30 mg·kg^−1^) dose‐dependently depressed mouse MV for the measured 40 min post‐administration (Figure 1 and Table S1). While a low dose of mitragynine (3 mg·kg^−1^) did not produce significant changes in respiration, a 10 mg·kg^−1^ dose induced significant and prolonged respiratory depression that was not further increased at higher doses (30 and 90 mg·kg^−1^) (Figure 1 and Table S1). These data suggest that the ability of mitragynine to induce respiratory depression is limited by a ceiling effect. Given that 7‐OH mitragynine is a more potent active metabolite of mitragynine that has already been suggested as the primary mediator of kratom‐induced effects (Kruegel et al., 2019), this ceiling effect may be due to a metabolic bottleneck (metabolic saturation) limiting mitragynine oxidation and accumulation of 7‐OH mitragynine.

**FIGURE 2:**
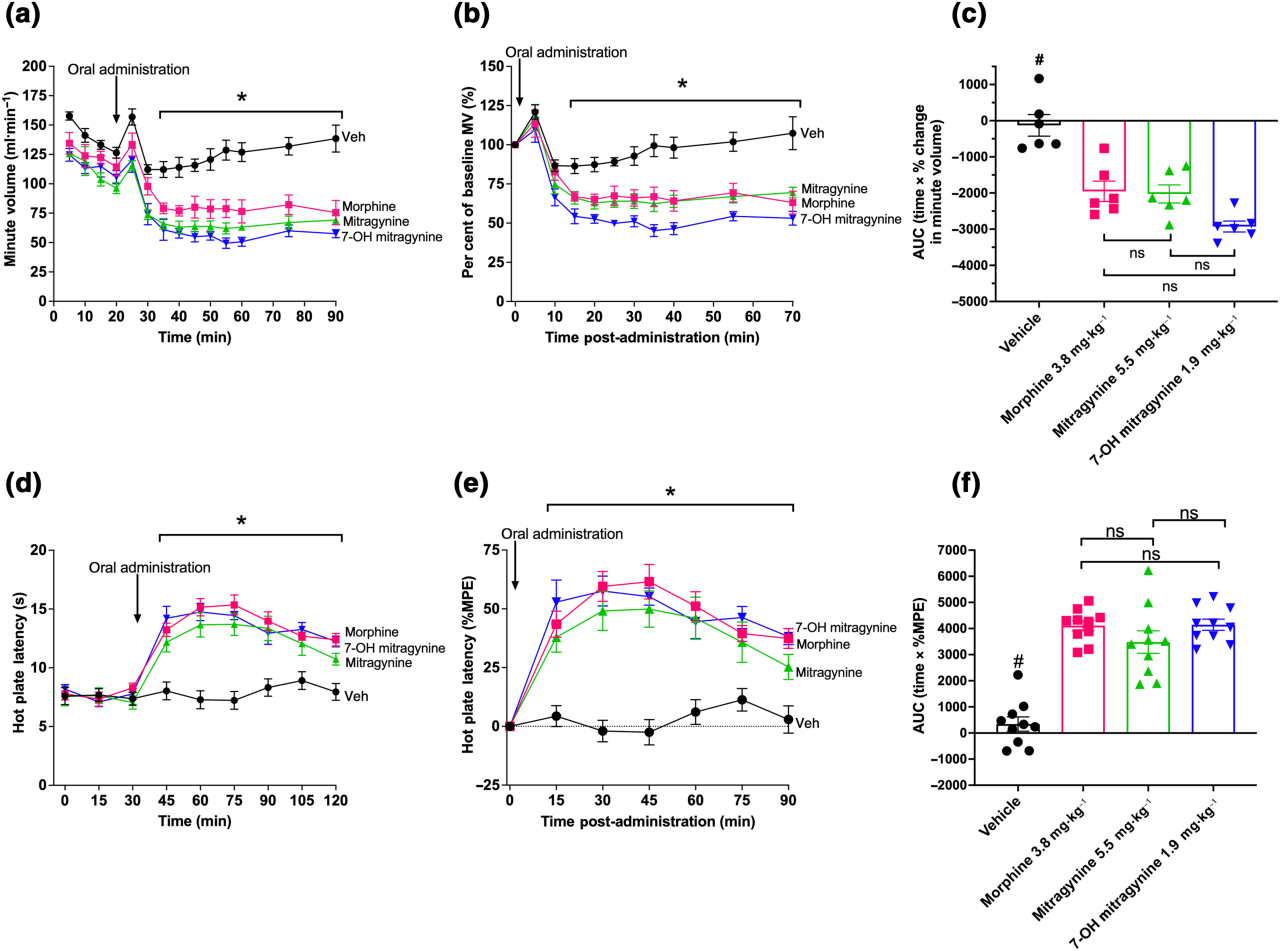
Equi‐respiratory depressant doses induce similar levels of anti‐nociception: (a,b) Orally administered doses of morphine (3.8 mg·kg^−1^), mitragynine (5.5 mg·kg^−1^) and 7‐OH mitragynine (1.9 mg·kg^−1^) all significantly depressed minute volume (MV) when compared with effects of vehicle alone. (c) There was no significant difference in the degree of respiratory depression induced by morphine, mitragynine and 7‐OH mitragynine. (d, e) The same doses of morphine, mitragynine and 7‐OH mitragynine induced significant increases in hot plate latency when compared with vehicle administered mice. (f) When comparing the overall degree of anti‐nociception induced by each drug, measured as AUC (time × %MPE), all drugs induced a significant and similar overall increase. *N* = 6 for (a)–(c) and *N* = 10 for (d)–(f). All drugs administered by oral gavage dosing. In (a), (b), (d) and (e), * *P* < 0.05, significantly different; ns, not significantly different, from vehicle; two‐way ANOVA with Dunnett's multiple comparisons. In (c) and (f), ^#^ *P* < 0.05, significantly different, ns, not significantly different, from opioid groups; one‐way ANOVA (Kruskal–Wallis) with Dunn's multiple comparison. See Table S1 for full test statistics

The maximum decrease in respiration (peak effect) induced by each drug and dose was used to estimate a dose for each opioid such that they would produce approximately the same degree of respiratory depression (Figure S1; see Section 2). Oral administration of calculated equi‐effective doses for mitragynine (5.5 mg·kg^−1^), 7‐OH mitragynine (1.9 mg·kg^−1^) and morphine (3.8 mg·kg^−1^) induced significant respiratory depression, and this was maintained for the entire period of observation (Figure 2a, b and Table S1). Administration of vehicle resulted in a transient decrease in mouse respiration (10–35 min post‐dose), but it was not significantly different from baseline following this period. Although 7‐OH mitragynine produced significantly more respiratory depression than morphine or mitragynine at some individual time points, AUC analysis of equi‐effective doses did not highlight any significant difference in the overall degree of respiratory depression induced over the entire observation period (Figure 2c and Table S1).

The equi‐effective doses of mitragynine, 7‐OH mitragynine and morphine in respiratory depression were then tested in hot plate anti‐nociception assays. All three agonists induced significant increases in mouse hot plate latency at all time points after opioid administration (90 min) (Figure 2d,e and Table S1). There were no significant differences at any time points between the three agonists, suggesting similar degrees of anti‐nociception and duration of action. AUC analysis of these data shows that the overall degree of anti‐nociception induced by each agonist was not significantly different from each other, but was significantly different from the effects of the vehicle (Figure 2f and Table S1).

### 3.2 Inhibition of CYP3A reduces mitragynine‐induced respiratory depression

The data above showing the ceiling effect of mitragynine in respiratory depression together with previous reports suggesting the need of metabolic conversion of mitragynine to 7‐OH mitragynine by CYP3A to induce anti‐nociception (Kruegel et al., 2019) led us to hypothesise that such metabolic conversion is also required to mediate the respiratory effects of mitragynine and underlies the built‐in ceiling for the effects of this compound. To address this hypothesis, we treated mice with ketoconazole (50 mg·kg^−1^, p.o.), a known inhibitor of CYP3A (Vermeer et al., 2016). Previous research has shown that a 30 min pre‐treatment with ketoconazole (35 mg·kg^−1^, p.o.) significantly inhibited the metabolic transformation of atorvastatin via CYP3A (Chang et al., 2014). Other work has demonstrated that a dose of 50 mg·kg^−1^ ketoconazole significantly inhibited loperamide metabolism while leaving P‐glycoprotein function unaffected (Seneca et al., 2010). Thus, a 30 min pretreatment with a dose of 50 mg·kg^−1^ p.o. was selected for these studies.

**FIGURE 3:**
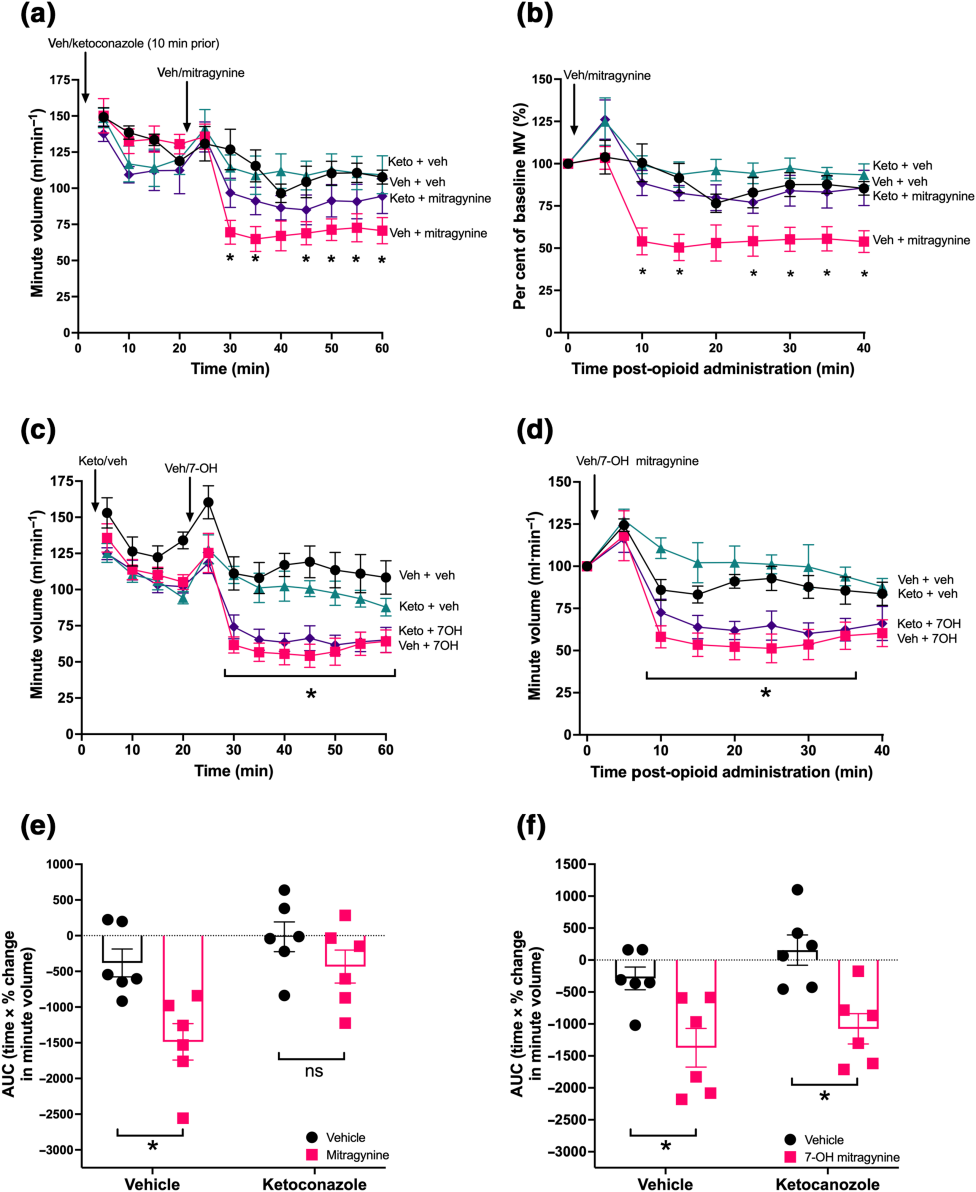
Effect of CYP3A inhibition on mitragynine and 7‐OH mitragynine induced respiratory depression: (a,b) Mitragynine (5.5 mg·kg^−1^) induced significant respiratory depression in mice pretreated with vehicle (veh) but not in mice pretreated with ketoconazole (keto) (50 mg·kg^−1^, 30 min), (c, d) 7‐OH mitragynine (1.9 mg·kg^−1^) induced significant respiratory depression in vehicle and ketoconazole (50 mg·kg^−1^) pretreated mice. Ketoconazole pretreatment significantly reduced overall respiratory depression induced by mitragynine (e) but not that induced by 7‐OH mitragynine (f). *N* = 6 for all groups. All drugs administered by oral gavage dosing. In (a) ‐ (d), * *P* < 0.05, significantly different from vehicle control or as indicated; two‐way ANOVA with Dunnett's multiple comparisons. In (e) and (f), * *P* < 0.05, significantly different; ns, not significantly different, as indicated; 2 × 2 factorial by two‐way ANOVA with Bonferroni's multiple comparisons. See Table S1 for full test statistics

While ketoconazole pretreatment seemed to produce a decrease in baseline respiration, this was not significantly different to the vehicle/control group (Figure S2). Pretreatment with ketoconazole significantly reduced mitragynine (5.5 mg·kg^−1^)‐induced respiratory depression (Figure 3a,b and Table S1). AUC analysis of these data shows that in mice pretreated with vehicle, mitragynine induced a significant degree of overall respiratory depression compared with the acute vehicle that was significantly reduced by ketoconazole pretreatment (Figures 3e and S3 and Table S1). This supports the hypothesis that the effects of mitragynine are likely to be mediated by its active metabolite.

However, as ketoconazole is known to affect other metabolic pathways other than CYP3A in rodents (Eagling et al., 1998), its effect on 7‐OH mitragynine was also investigated as a control. Mice administered 7‐OH mitragynine (1.9 mg·kg^−1^) showed significant respiratory depression at all time points following administration in both vehicle and ketoconazole‐pretreated mice (Figure 3c,d and Table S1), though when these data were analysed as percentage of baseline respiration, neither group produced significant respiratory depression at the final time point (40 min) compared with vehicle control (Figure 3d and Table S1). Further analysis by AUC demonstrated that not only did 7‐OH mitragynine depress respiration in both vehicle and ketoconazole pretreated mice but also that the degree of respiratory depression induced by 7‐OH mitragynine was not significantly different between pretreatments (Figures 3f and S3B and Table S1).

**FIGURE 4:**
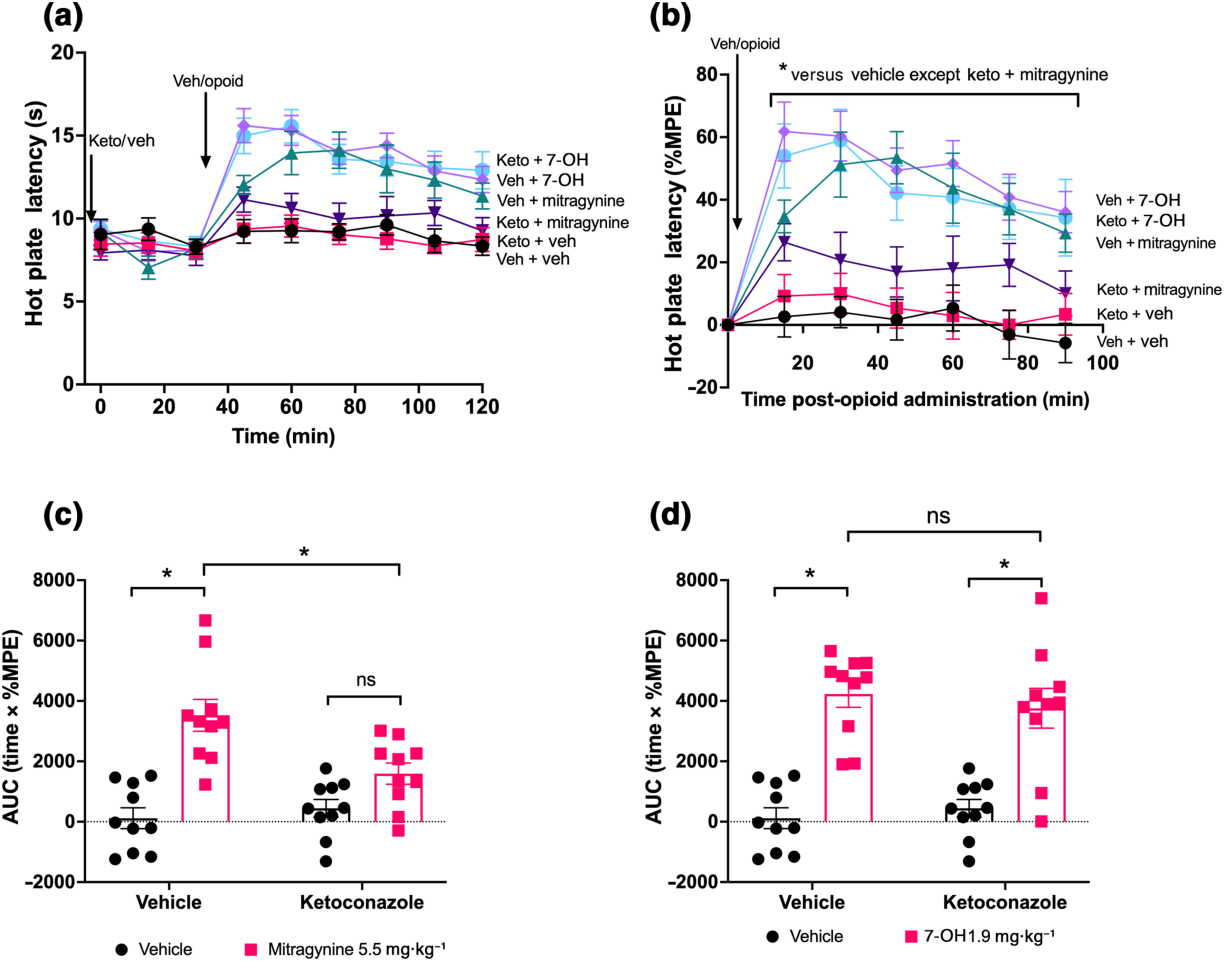
Inhibition of CYP3A by ketoconazole pretreatment prevents mitragynine but not 7‐OH mitragynine‐induced anti‐nociception: (a, b) 7‐OH mitragynine (1.9 mg·kg^−1^) and mitragynine (5.5 mg·kg^−1^) induced significant anti‐nociception in vehicle‐pretreated mice; however, only 7‐OH induced significant anti‐nociception in ketoconazole‐pretreated mice. (c, d) 7‐OH mitragynine induced the same degree of anti‐nociception in both vehicle‐ and ketoconazole‐pretreated mice whereas mitragynine was significantly reduced by ketoconazole treatment as well as not being significant from vehicle. *N* = 10 for all groups. All drugs administered by oral gavage dosing. In (b), * *P* < 0.05, significantly different as indicated; two‐way ANOVA with Dunnett's multiple comparisons. In (c) and (d), * *P* < 0.05, significantly different, ns not significantly different, as indicated; 2 × 2 factorial by two‐way ANOVA with Bonferroni's multiple comparisons. See Table S1 for full test statistics

We obtained similar results when we assessed the effects of ketoconazole pretreatment on mitragynine and 7‐OH mitragynine‐induced anti‐nociception. Both mitragynine and 7‐OH mitragynine induced significant increases in hot plate latency compared with vehicle (at all time points) for mice that received vehicle pretreatment (Figure 4a,b and Table S1). In ketoconazole‐pretreated mice, 7‐OH mitragynine, but not mitragynine, induced a significant and prolonged increase in hot plate latency at any time point over the observation period (Figure 4b and Table S1). AUC analysis of these data further demonstrated that in mice pretreated with vehicle or ketoconazole, 7‐OH mitragynine induced a significant degree of anti‐nociception compared with the acute vehicle (Figure 4d and Table S1), whereas in ketoconazole pretreated mice, mitragynine failed to induce significant anti‐nociception compared with acute vehicle (Figure 4c and Table S1).

## 4 DISCUSSION

In this study, we report the respiratory depressant effects of the kratom alkaloid mitragynine and its oxidised derivative 7‐OH mitragynine. We also showed that the respiratory depressant effects of mitragynine were partly due to its metabolic conversion to 7‐OH mitragynine by CYP3A. These results are in line with previous reports suggesting that mitragynine‐induced anti‐nociception can be mediated by its active metabolite (Kruegel et al., 2019).

Available evidence suggests that mitragynine, when administered orally, has an improved side‐effect profile, compared with typical opioids such as morphine or codeine, with reports showing reduced respiratory depression (Macko et al., 1972) and GI transit inhibition, although some signs of withdrawal have been reported (Hassan et al., 2021; Yusoff et al., 2016). Altogether, these data suggest that mitragynine may exhibit a greater safety margin for overdose compared with other opioids. Mitragynine has been associated with both low toxicity in mice (LD_50_ > 400 mg·kg^−1^) (Sabetghadam et al., 2013; Smith et al., 2019) and an extremely low toxicity compared with other opioids in humans (44 total vs. ~50,000 all opioid deaths 2017) (NIDA, 2018; Veltri & Grundmann, 2019). The prevalence of kratom as an increasingly sought‐after alternative to traditional opioid antagonist/agonist combinations such as suboxone or the replacement opioid methadone has suggested a perceived benefit in terms of intoxication, overdose protection or withdrawal management, either individually or in combination (Grundmann, 2017; Veltri & Grundmann, 2019).

The mechanism underlying the improved safety profile for mitragynine has been suggested to be ‘metabolic saturation’, namely, when the generation of the active metabolite of the drug is limited by the rate of conversion of the enzyme(s) responsible for its metabolism. As such, the extent of induction of opioidergic effects by mitragynine will be determined by its conversion to 7‐OH mitragynine by CYP3A (Kruegel et al., 2019). This would explain the built‐in ceiling effect observed in this study, where doses of mitragynine above 10 mg·kg^−1^ produce the same respiratory depressant effects. This hypothesis is also supported by the experiments using the CYP3A inhibitor ketoconazole at a dose that has been shown to modulate CYP3A opioid metabolism (Chakraborty, Uprety, Slocum, et al., 2021; Seneca et al., 2010), which inhibited the effects of mitragynine but not those of 7‐OH mitragynine. Importantly, the effects observed in respiratory depression measurements are mirrored in anti‐nociceptive measurements. However, the possibility that ketoconazole affects differently the absorption rates of mitragynine and 7‐OH mitragynine and the pharmacodynamic profile of ketoconazole as it relates to opioid receptors remains to be further investigated.

In the context of metabolic conversion, mitragynine could be compared with codeine whose conversion into morphine (and induction of opioidergic effects) depends on the enzyme CYP2D6. It could be anticipated that, as with codeine, individuals with genetic mutations leading to alterations in the levels of activity of its metabolising enzyme would have different sensitivities to the effects of kratom. From a drug discovery standpoint, however, this metabolic regulation of opioidergic effects offers significant potential to improve safety profiles of opioid agonists.

The interaction between opioids and their metabolic enzymes is also likely to be altered in polydrug exposure either with prescription or illicit drugs (Darke, 2003). In particular, the combination of benzodiazepines and opioids is known to be extremely dangerous, not only through the combined respiratory depressant effects of both drugs but also because benzodiazepines can act as substrates for cytochrome P450 enzymes (Gudin, 2012; Smith, 2009). This has been postulated to be responsible for altering the pharmacodynamics of oxycodone and enhancing its potential lethality (Ji et al., 2021). Similarly, barbiturates (e.g., phenobarbital and pentabarbital) and antibiotics, such as rifampicin, are strong inducers of CYP3A enzymes and thus, when used in combination, would be expected to increase the effects of mitragynine. Therefore, as with other opioids, the safety profile of mitragynine and its metabolites must also be considered in a polydrug context in guiding dosage adjustment in potential cases of poly therapy.

Taking into consideration that mitragynine itself has been shown to bind and activate μ receptors (Kruegel et al., 2016), albeit with lower efficacy than 7‐OH mitragynine, it is possible that some of its actions might be directly mediated by mitragynine. It would then follow that the overall effect of mitragynine is likely to result from the activation of μ receptors in the presence of two ligands with different efficacies, whereby binding of a lower efficacy ligand such as mitragynine will decrease the response of a higher efficacy agonist such as 7‐OH mitragynine. This could function alongside metabolic saturation as a mechanism that limits the effects of mitragynine.

A recent study has also demonstrated that 7‐OH mitragynine can be metabolised further to mitragynine pseudoindoxyl in a manner that is specific to human plasma when compared with primates, rodents and dogs (Kamble et al., 2020). Although the exact metabolic pathway responsible for this conversion is yet to be elucidated, mitragynine pseudoindoxyl has been reported to have greater efficacy at μ receptors than 7‐OH mitragynine, as well as being a δ receptor antagonist (Varadi et al., 2016). Thus, it is likely that following human consumption of mitragynine (kratom), the overall physiological response will be induced by multiple mitragynine alkaloids with the metabolic rate conversion playing a key role in the observed response (Chakraborty, Uprety, Slocum, et al., 2021). Further studies should aim to establish the relative intrinsic efficacy of these atypical opioids to assess whether their effects, in vivo, reflect such efficacies.

Two interesting comparisons can be made between the actions of mitragynine and codeine and oxycodone. Codeine is a relatively safe opioid in the management of moderate pain with reduced respiratory liability, though it still presents a significant risk in terms of dependence and addiction (Nielsen et al., 2018). In contrast, management of moderate to severe pain using oxycodone has been associated with significant numbers of overdose deaths over the last 10–15 years (Kibaly et al., 2021). Interestingly, both agonists are metabolised by cytochrome P450 enzymes with codeine primarily metabolised by CYP2D6 (Crews et al., 2021) and oxycodone metabolised by both CYP2D6 and CYP3A (Umukoro et al., 2021). Codeine may become less safe following the induction of CYP2D6 activity, because of the formation of morphine as an active metabolite (Crews et al., 2021; Haertter, 2013). Conversely, inhibition of CYP3A metabolism may cause a delay in the clearance of oxycodone, exacerbating its opioidergic side effects (Rytkönen et al., 2020; Umukoro et al., 2021). In the case of mitragynine, the results presented here suggest that induction of the CYP3A metabolic pathway may increase the chance of toxicity being achieved with kratom, due to enhanced formation of 7‐OH mitragynine.

Of note, the experiments presented here assessed respiratory depression in mice breathing air to facilitate comparisons with previous studies of novel opioid agonists. While opioids do directly inhibit neurons responsible for respiratory rhythmogenesis (Saunders & Levitt, 2020), they are also responsible for inhibiting the hypercapnic reflex to stimulate breathing (Palkovic et al., 2020) and thus further exacerbate respiratory distress and increase the likelihood of a fatal overdose. Previous research has shown that the potency of an opioid to depress respiration is enhanced under hypercapnic conditions (Czapla & Zadina, 2005), suggesting that a progressively worsening feedback loop may occur during overdose. The effects of hypercapnic challenges on mitragynine‐induced respiratory depression will be an important future step in defining its full safety profile, particularly with regard to escalating concentrations of CO_2_ that aim to mimic the progressively deteriorating respiratory pathology of an overdose.

In summary, our results suggest that the respiratory depressant effects of mitragynine are likely to be mediated by the generation of the active metabolite 7‐OH mitragynine. The metabolic saturation of the enzymatic oxidation of mitragynine provides a built‐in ceiling for respiratory depression that, together with the very weak partial agonist activity displayed by mitragynine when bound to μ receptors (acting to reduce the effects of 7‐OH mitragynine), may underlie its improved safety profile as an analgesic.

## AUTHOR CONTRIBUTIONS

RH, JRL and MC designed experiments. RH performed experiments and analysed the data. RH, JRL and MC wrote the manuscript. ACK and JAJ provided reagents. All authors edited and reviewed the manuscript.

## CONFLICT OF INTEREST

ACK is a co‐founder and shareholder of Kures, Inc. (a subsidiary of ATAI Life Sciences) and Gilgamesh Pharmaceuticals, Inc. Kures is currently developing a mitragynine analogue as a potential pharmaceutical. ACK is named as an inventor on mitragynine‐related patent applications (WO 2020/160280 A1, WO 2020/037136 A1, US Patent 10,961,244 B2) owned by Columbia University and Kures. JAJ is a co‐founder of Kures and named as an inventor on mitragynine‐related patents (WO 2020/160280 A1, WO 2020/037136 A1, US Patent 10,961,244 B2) owned by Columbia University. No funding was provided by Kures for this work.

## DECLARATION OF TRANSPARENCY AND SCIENTIFIC RIGOUR

This Declaration acknowledges that this paper adheres to the principles for transparent reporting and scientific rigour of preclinical research as stated in the *BJP* guidelines for Design and Analysis, and Animal Experimentation, and as recommended by funding agencies, publishers and other organisations engaged with supporting research.

## Supporting information


**Table S1.** Figure statistics

**Figure S1.** Linear regression to derive ~40% (indicated with dashed lines) depressant doses of each opioid.

**Figure S2.** **Baseline respiration was not significantly affected by ketoconazole pre‐treatment. A**) Baseline MV only. **B**) Area under the curve analysis (AUC time x % change in MV) shows no significant overall effect of ketoconazole pre‐treatment on baseline respiration. Comparisons made in a 2x2 factorial by Two‐way ANOVA with Bonferroni's multiple comparisons in **B**. ns = not significant as indicated. N = 6 for all groups.

**Figure S3.** **Inhibition of CYP3A by ketoconazole pre‐treatment prevents mitragynine but not 7‐OH mitragynine respiratory depression**. Ketoconazole (50 mg/kg) pre‐treatment significantly attenuates mitragynine (5.5 mg/kg) respiratory depression (**A**) but not 7‐OH mitragynine (1.9 mg/kg) respiratory depression (**B**). Comparisons made in a 2x2 factorial by Two‐way ANOVA with Bonferroni's multiple comparisons in **A and B**. * indicates p < 0.05 vs vehicle control or as indicated. ns = not significant. N = 6 for all groups.

## Data Availability

The data that support the findings of this study are available from the corresponding authors upon reasonable request.
