# Supplementary material for: The respiratory depressant effects of mitragynine are limited by its conversion to 7‐OH mitragynine
Source: Br J Pharmacol. 2022 Mar 30;179(14):3875–85. doi: 10.1111/bph.15832 (PMC9314834; doi:10.1111/bph.15832)
Supplement: Supplementary file 1 — Table S1. Figure statistics [file BPH-179-3875-s004.docx]

**Supplementary Table 1 – Figure statistics**

| **Figure** | **Panel** | **Test** | **Details (F(DFn,DFd)** |
| --- | --- | --- | --- |
| Figure 1 | **C** | One-way ANOVA (Kruskal-Wallis) with Dunn’s multiple comparison | Approx. P value = 0.0001, Kruskal Wallis stat = 23.13, DFn-4 DFd-30 |
|  | **F** |  | Approx. P value = 0.0003, Kruskal Wallis stat = 18.65, DFn-3 DFd-24 |
|  | **I** |  | Approx. P value = 0.0002, Kruskal Wallis stat = 20.13, DFn-3 DFd-24 |
| Figure 2 | **A** | Two-way ANOVA with Dunnett’s multiple comparisons | Interaction: F (39, 280) = 2.056, p=0.0005  Row: F (13, 280) = 42.07, p<0.0001  Column: F (3, 280) = 164.1, p<0.0001 |
|  | **B** |  | Interaction: F (30, 220) = 2.442, p=0.0001  Row: F (10, 220) = 38.05, p<0.0001  Column: F (3, 220) = 89.96, p<0.0001 |
|  | **C** | One-way ANOVA (Kruskal-Wallis) with Dunn’s multiple comparison | Approx. P value = 0.0005, Kruskal Wallis stat = 17.77, DfN-3 DfD-24 |
|  | **D** | Two-way ANOVA with Dunnett’s multiple comparisons | Interaction: F (24, 324) = 4.672, p<0.0001  Row: F (8, 324) = 41.16, p<0.0001  Column: F (3, 324) = 62.26, p<0.0001 |
|  | **E** |  | Interaction: F (28, 252) = 3.300, p<0.0001  Row: F (6, 252) = 25.03, p<0.0001  Column: F (3, 252) = 73.74, p<0.0001 |
|  | **F** | One-way ANOVA (Kruskal-Wallis) with Dunn’s multiple comparison | Approx. P value < 0.0001, Kruskal Wallis stat = 23.44, DfN-3 DfD-40 |
| Figure 3 | **A** | Two-way ANOVA with Dunnett’s multiple comparisons | Interaction: F (33, 240) = 1.633, p=0.0203  Row: F (11, 240) = 14.85, p<0.0001  Column: F (3, 240) = 14.96, p<0.0001 |
|  | **B** |  | Interaction: F (24, 180) = 1.155, p=0.2901  Row: F (8, 180) = 11.62, p<0.0001  Column: F (3, 180) = 35.59, p<0.0001 |
|  | **C** |  | Interaction: F (33, 240) = 1.948, p=0.0024  Row: F (11, 240) = 23.86, p<0.0001  Column: F (3, 240) = 61.94, p<0.0001 |
|  | **D** |  | Interaction: F (24, 180) = 1.459, p=0.0864  Row: F (8, 180) = 18.62, p<0.0001  Column: F (3, 180) = 43.30, p<0.0001 |
|  | **E** | Two-way ANOVA with Bonferroni’s multiple comparisons | Interaction: F (1, 20) = 2.731, p=0.1392  Row: F (1, 20) = 10.10, p=0.0047  Column: F (1, 20) = 11.61, p=0.0028 |
|  | **F** |  | Interaction: F (1, 20) = 0.09138, p=0.7656  Row: F (1, 20) = 2.317, p=0.1436  Column: F (1, 20) = 22.75, p=0.0001 |
| Figure 4 | **B** | Two-way ANOVA with Dunnett’s multiple comparisons | Interaction: F (30, 378) = 1.605, p=0.0250  Row: F (6, 378) = 14.56, p<0.0001  Column: F (5, 378) = 42.91, p<0.0001 |
|  | **C** | Two-way ANOVA with Bonferroni’s multiple comparisons | Interaction: F (1, 36) = 8.470, p=0.0062  Row: F (1, 36) = 4.247, p=0.0466  Column: F (1, 36) = 34.33, p<0.0001 |
|  | **D** | Two-way ANOVA with Bonferroni’s multiple comparisons | Interaction: F (1, 36) = 0.7758, p=0.3843  Row: F (1, 36) = 0.02450, p=0.8765  Column: F (1, 36) = 66.29, p<0.0001 |
