# Supplementary figures and images for: The respiratory depressant effects of mitragynine are limited by its conversion to 7‐OH mitragynine
Source: Br J Pharmacol. 2022 Mar 30;179(14):3875–85. doi: 10.1111/bph.15832 (PMC9314834; doi:10.1111/bph.15832)

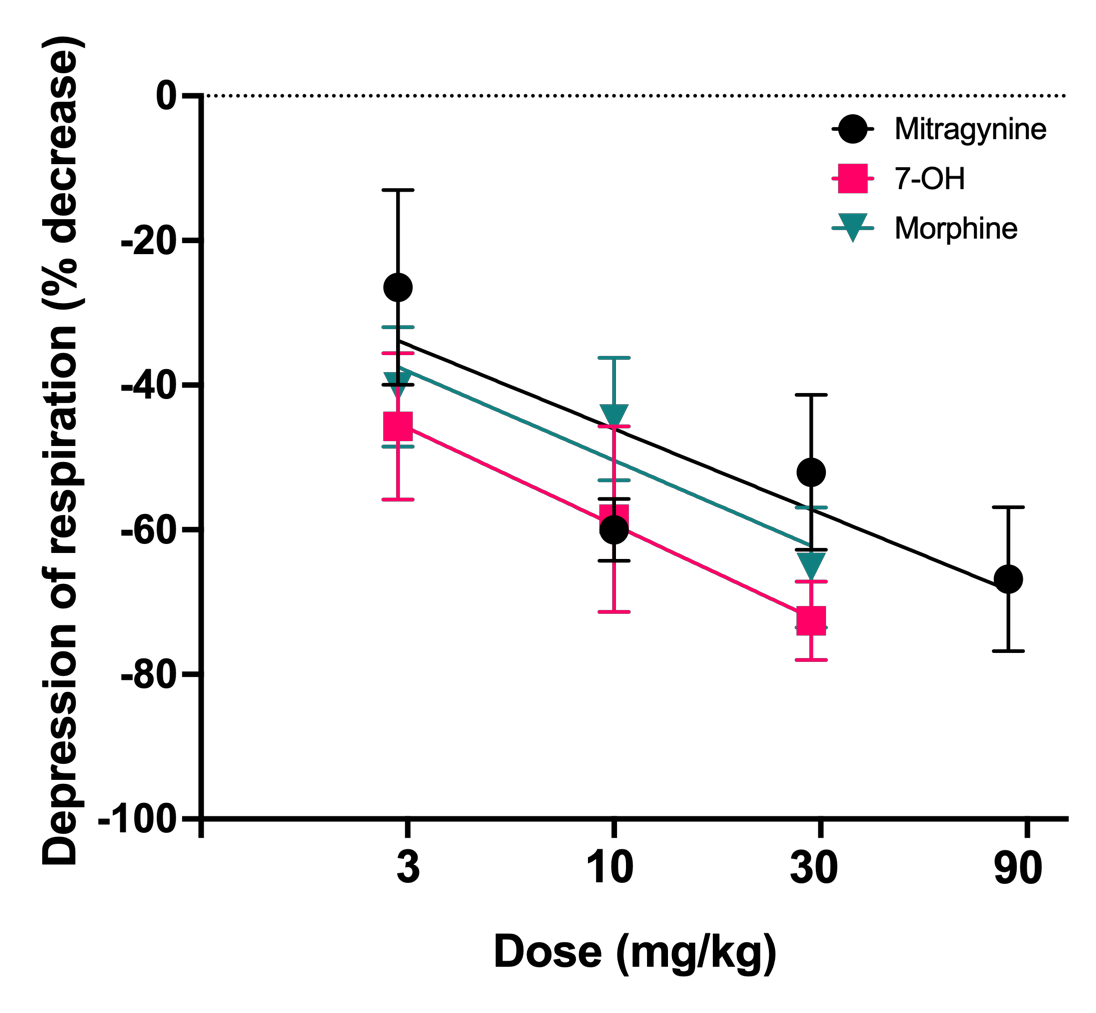

Supplement: Supplementary file 2 — Figure S1. Linear regression to derive ~40% (indicated with dashed lines) depressant doses of each opioid. [file BPH-179-3875-s003.tiff]

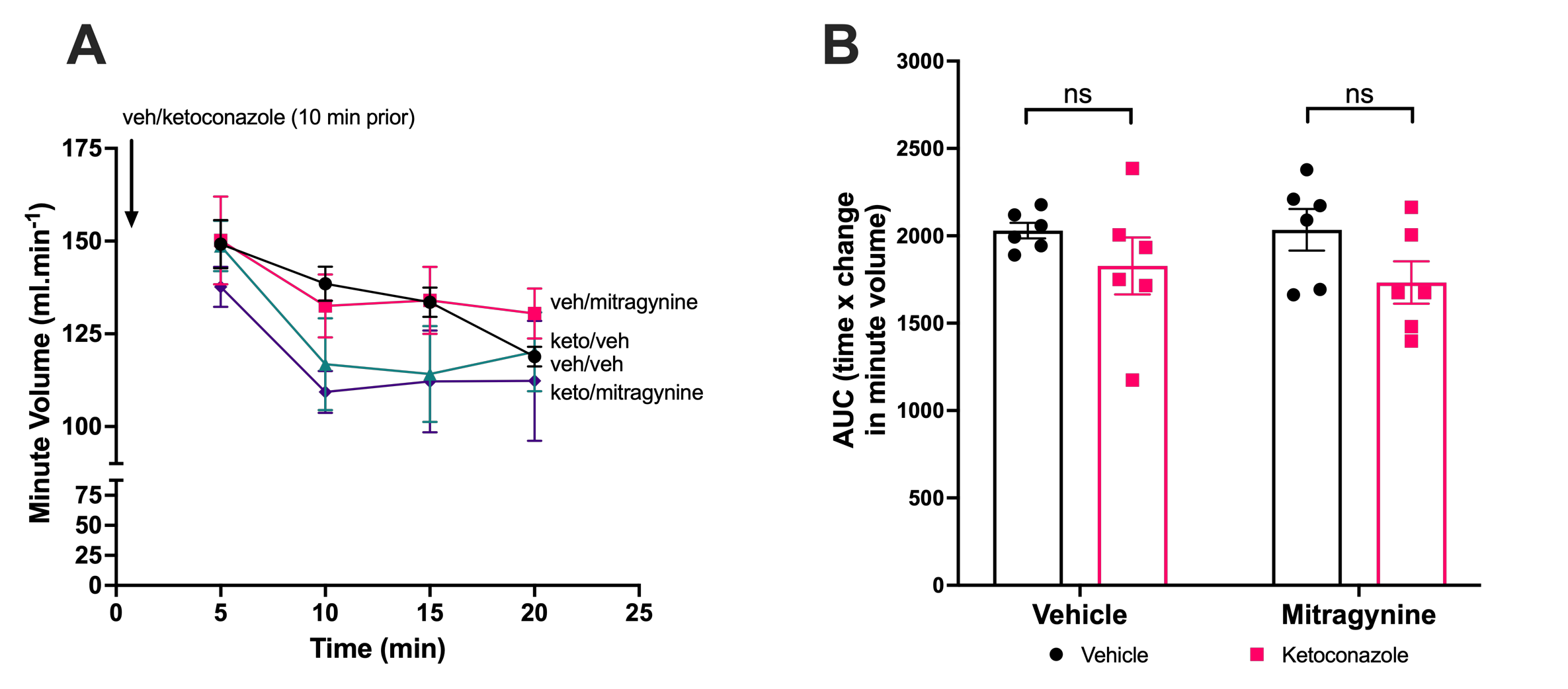

Supplement: Supplementary file 3 — Figure S2. Baseline respiration was not significantly affected by ketoconazole pre‐treatment. A) Baseline MV only. B) Area under the curve analysis (AUC time x % change in MV) shows no significant overall effect of ketoconazole pre‐treatment on baseline respiration. Comparisons made in a 2x2 factorial by Two‐way ANOVA with Bonferroni's multiple comparisons in B. ns = not significant as indicated. N = 6 for all groups. [file BPH-179-3875-s001.tiff]

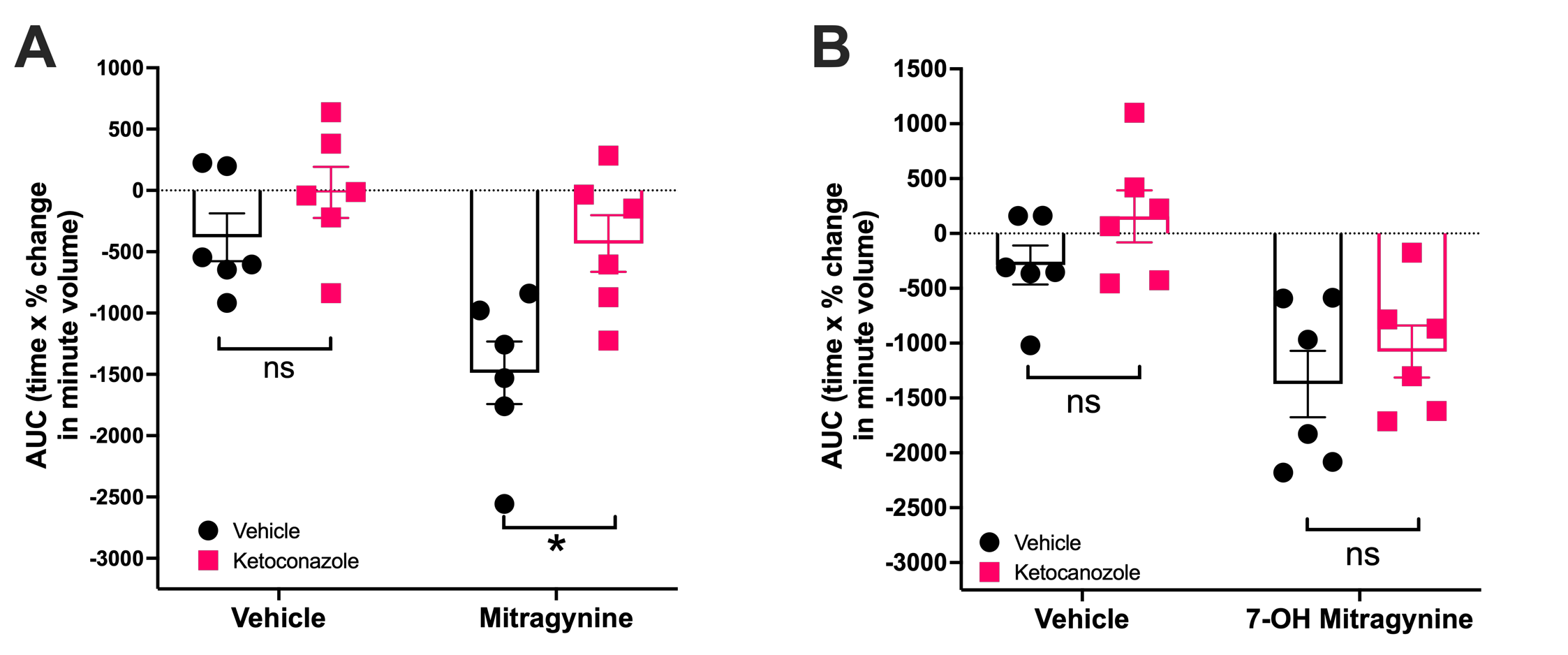

Supplement: Supplementary file 4 — Figure S3. Inhibition of CYP3A by ketoconazole pre‐treatment prevents mitragynine but not 7‐OH mitragynine respiratory depression. Ketoconazole (50 mg/kg) pre‐treatment significantly attenuates mitragynine (5.5 mg/kg) respiratory depression (A) but not 7‐OH mitragynine (1.9 mg/kg) respiratory depression (B). Comparisons made in a 2x2 factorial by Two‐way ANOVA with Bonferroni's multiple comparisons in A and B. * indicates p < 0.05 vs vehicle control or as indicated. ns = not significant. N = 6 for all groups. [file BPH-179-3875-s002.tiff]
